# Understanding modifiable barriers to human milk donation in the United Kingdom

**DOI:** 10.1111/jhn.13405

**Published:** 2024-12-01

**Authors:** Amy Brown, Sara Jones, Catrin Griffiths, Wendy Jones, Gillian Weaver, Natalie Shenker

**Affiliations:** ^1^ Centre for Lactation, Infant Feeding and Translation (LIFT) Swansea University Swansea UK; ^2^ Faculty Medicine, Health and Life Sciences Swansea University Swansea UK; ^3^ The Human Milk Foundation, Gossams End Berkhamsted UK; ^4^ Department of Surgery and Cancer Imperial College London London UK

**Keywords:** breastfeeding, donor human milk, health service delivery, infant feeding services, milk banking infrastructure, milk donation

## Abstract

**Background:**

When premature infants cannot receive their own mother's milk, donor human milk (DHM) is the first‐line recommended option, with growing demand for DHM use outside of neonatal units. To meet the potential need, we need to consider whether DHM supply can increase. This study aimed to explore the reasons that prevent women who wish to donate their milk in the United Kingdom from doing so to understand which barriers may be modifiable.

**Methods:**

Women who wanted to donate their milk but did not do so completed an online survey. Open and closed questions examined the response they received, their reasons for not donating and what they did with any milk that they had already stored.

**Results:**

Out of 732 mothers, 391 (53.4%) did not enquire as they did not think it was possible for them, 218 (29.8%) enquired but were told that they could not donate, 59 (8.1%) enquired but decided not to proceed and 64 (8.7%) received no response. Reasons for being told they could not donate included the use of certain medications, infant age, inadequate staffing, geographic barriers and incorrect storage. Process aspects (e.g., blood tests, practicalities) and lifestyle limitations led mothers to decide not to donate.

**Conclusions:**

Although some women will be prevented from donating due to medication or health issues, investment in milk banking staffing and infrastructure and awareness campaigns could increase DHM supply, enabling guidelines to extend eligibility criteria for receiving DHM such as for late preterm infants, gestational diabetes or to support low maternal milk supply.

## Key points

- Many more women want to be able to donate their milk than do so. For some, milk donation will not be possible, but many barriers are modifiable.
- Increased funding to strengthen infrastructure and staffing would enable more women to be able to donate their milk.
- Earlier information and awareness around milk donation would support women to start the process at an earlier stage and to follow guidelines, increasing numbers enroled and volumes donated.

## BACKGROUND

When premature infants cannot receive their mother's own milk (MOM), donor human milk (DHM) can help protect their health and development, reducing the risk of complications associated with prematurity^1^, ^2^ and healthcare costs.^3^, ^4^ DHM can also support parental well‐being, through reducing anxiety around infant health and milk production,^5^, ^6^, ^7^, ^8^ and can be supportive of maternal lactation when used temporarily as a bridge to the establishment of maternal milk supply as part of an optimal lactation support programme.^9^, ^10^

UK guidelines currently recommend DHM is considered for premature infants born at <32 weeks gestation,^11^ and the use of DHM in hospitals for this population is increasing and expected to increase further due to parental preference, clinical trials and improved evidence.^12^ Limited DHM has also been used for moderate or late preterm infants in hospitals and in community settings to support low milk supply, for older infants with health complications and when breastfeeding is not possible due to reasons such as maternal cancer^13^ and could play an increasing role in replacing formula as the first‐line supplementation for other patient groups in the future.

Although some parents have hesitation over donor milk, research suggests that some would like the option if mothers' own milk needs to be supplemented. In one interview study with 24 mothers of term infants in the United States, 56% said that if needed they would want the option to supplement with DHM rather than formula milk,^14^ which is in accordance with unpublished data from a recent survey by the charity the Human Milk Foundation in the United Kingdom. On the other side of the milk donation relationship, mothers have expressed that the act of donation can also be a positive experience for mothers,^15^ especially after infant loss.^16^ However, wanting to but not being able to donate can be distressing, especially for those who have experienced breastfeeding difficulties, have postnatal depression or have experienced infant loss.^17^, ^18^ Questions therefore arise around how more infants could potentially receive DHM supplementation and how milk banking services could increase to meet the growing need.

One aspect central to this is how the number of women able to donate milk can be increased. To answer this question, we need to better understand barriers and disparities in milk donation. Smaller scale, often qualitative research outside of the United Kingdom has identified barriers to donation including time, milk storage guidelines and difficulties transporting milk which dissuade, prevent or decrease milk donations.^19^, ^20^, ^21^, ^22^ However, little is known about barriers to milk donation in the United Kingdom, which has a different milk banking infrastructure to regions such as the United States, or who may be more likely to face disparities in donation. The aim of our current study was therefore to explore the experiences of women who wanted to donate their milk in the United Kingdom but were unable to do so. This included investigating the reasons mothers could not donate, their feelings about being unable to donate and what happened to the milk if unable to donate.

## METHODS

### Participants

This paper reports findings from a larger study exploring experiences of milk donation in the United Kingdom. Participants in this analysis consisted of those who wanted to donate their breast milk but did not do so, either because they thought they would not be eligible, were told that they were not eligible, decided not to after enquiry or did not receive a response. No time since wanting to donate was set as we wanted insight into the time frame of memories and the impact upon donation. We recognise the potential recall bias of this decision and apply caution to those recalling experiences from a longer duration. Further inclusion criteria were age 16+, able to complete the survey in the English language and able to give informed consent.

Mothers from across the United Kingdom could take part and we used an internet sampling approach to reach mothers across the nation. In the United Kingdom, there are geographical disparities in who can donate milk because of the location of milk banks and milk bank hubs (smaller units that typically collect donor milk and store larger volumes of DHM but do not process milk). There are 14 milk banks across the United Kingdom, ranging from single hospital services to those that support a whole country (e.g., Scotland) or region (see https://ukamb.org/milk-banks). There are also an increasing number of hubs that enable women to donate milk, including those for the Hearts Milk Bank including Swansea in South Wales, Norfolk, Sussex, Kent and Northumbria, with the aim of widening access to donation.

Full ethical permission for the study was gained from Swansea University School of Health and Social Care Research Ethics Committee. Participants gave informed consent, and all aspects of the study were carried out in line with the Declaration of Helsinki. All participants gave informed consent prior to completing the survey.

### Measures

Participants completed an online survey hosted via Qualtrics consisting of a series of tick box, Likert scale (5‐point scale, strongly agree to strongly disagree) and open‐ended questions. The relevant questions for this analysis include:

- Demographic details (age, education, location).
- Where enquiries were made to donate and when.
- Why donation was not possible.
- Use and disposal of already expressed breast milk.

### Procedure

Data were collected between May 2022 and March 2023. The study was first conducted in Wales (May–September 2022) specifically because Wales did not have its own milk bank or hub before 2021, meaning there were significant regional disparities in donations. Oversampling in this region enabled us to explore the experiences of the subgroup who were not able to donate due to a lack of infrastructure. Participation was then opened up across the United Kingdom (November 2022–March 2023). This meant that a higher proportion of participants (23.4%) were from Wales compared to the proportion of the UK population who live in Wales (4.6%).

Study adverts were placed on social media by the team with details of the study. Posts were shared on the academic/organisational pages of the research team, including Instagram, Facebook pages and Twitter, with encouragement for interested viewers and organisations to share further. The adverts were shared at least 200 times over the data collection period (privacy settings prevent a specific number from being calculated), with metrics suggesting a post reach of at least 250,000 accounts. If potential participants were interested in finding out more, they clicked on a link to take them to the participant information sheet and consent form. If inclusion criteria were met and consent given, the full survey loaded. Once completed, a debrief statement was given, explaining the study, thanking them for participation and giving them contact details for support organisations if needed.

### Data analysis

Data were analysed using SPSS version 29. To increase the reliability of an online survey against fraudulent or bot activity, we included several steps. It was only possible for one entry to be submitted per IP address. No financial incentives were used which appears to reduce false entries. Qualtrics collects time stamp data and time taken to complete the survey. These entries were checked to ensure responses were not of a very short duration or numerous responses submitted in a close time period, which may be indicative of bot completions. We also included several open‐ended text questions which were checked for unusual or identical responses. These questions also asked for specific information that may be more difficult for bots to complete, such as specific milk bank, time period and postcode, which we checked and matched. We were confident that responses appeared genuine.

Descriptive statistics were then used to compute score frequencies, followed by a series of inferential tests. For experiences, data participants responded to barriers via a five‐point Likert scale, strongly agree to strongly disagree. We present the combined data for those who strongly agreed or agreed with each statement to show the strength of the barriers. For interval data, to compare differences in experiences (such as the reason for not donating) between demographic groups (e.g., age, education, location), we used multivariate analysis of variance. Where data were nominal, we used *χ* ^2^ to explore associations between demographic background and experiences.

Qualitative data from the open‐ended questions were analysed through a simple content analysis approach (e.g., counting mentions of a certain reason for not being able to donate). Author C. G. initially coded the data, and to enhance the trustworthiness of the data, author AB then reviewed the proposed themes. Where disagreement occurred, themes were discussed until agreed.

## RESULTS

Overall, 732 mothers thought about donating their milk but did not do so. Within this cohort, 391 (53.4%) did not enquire about donation as they did not think it was possible, while 341 (46.6%) enquired but did not go on to donate. Of those who enquired but did not donate, 218 (63.9%) were told that they could not, 59 (17.3%) decided not to proceed and 64 (18.8%) did not get a response. Full demographic details of the sample are shown in Table 1.

**Table 1:** Participant demographic background.

Category	Subcategory	*N*	%
Age	18–24	31	4.2
	25–29	99	13.5
	30–34	267	36.5
	35–39	227	31.0
	40–44	79	10.8
	45+	29	4.0
Education	No formal qualifications	0	0.0
	GCSE or equivalent	21	2.9
	A level or equivalent	97	13.3
	Degree or equivalent	309	42.2
	Postgraduate qualification or equivalent	302	41.3
	Prefer not to say or missing	3	0.4
Ethnicity	Asian or Asian British: Bangladeshi	2	0.3
	Asian or Asian British: Chinese	3	0.4
	Asian or Asian British: Indian	8	1.1
	Asian or Asian British: Pakistani	1	0.1
	Any other Asian background	2	0.3
	Black or Black British	10	1.3
	Mixed or Multiple	20	2.7
	White British or Irish	636	86.8
	White (other)	44	6.0
	Gypsy or Irish traveller	2	0.3
	Any other group	1	0.1
	Prefer not to say	3	0.4
Number of children	First‐time mother	351	48.0
	Second time or more mother	381	52.0
Country	England	495	67.6
	Northern Ireland	17	2.3
	Scotland	49	6.7
	Wales	171	23.4

Abbreviation: GCSE, general certificate of secondary education.

Participants recalled wanting to donate milk from 2002 to 2023, with the majority (n = 698, 95.4%) describing experiences within the 2018–2023 period. Nineteen participants (2.6%) reported attempting to donate over two or more separate time periods with different babies. Where participants recalled two instances, we reported their most recent experience of attempting to donate.

### Demographic differences in response to donation request

Differences in demographics were compared between the four groups (told no, decided no, no response and did not ask). A *χ* ^2^ test found a significant association between the education group and response [*χ* ^2^ = 8.40, *p* = 0.038]. Compared to those with secondary education, those with a degree award or above were more likely to be told no (24.6% vs. 30.9%) or decide no (3.4% vs. 9.0%). Those with secondary education were less likely to enquire about donation (62.7% vs. 51.5%) compared with women with a higher level of education.

A *χ* ^2^ found significant association was also found for the country [*χ* ^2^ = 58.15, *p* = <0.001]. Examining response by country:

- *Told no*: Northern Ireland (64.7%), England (31.7%), Scotland (24.5%), Wales (22.2%).
- *Decided no*: Scotland (30.6%), Northern Ireland (11.8%), England (7.1%), Wales (4.1%).
- *No response*: England (9.3%), Wales (9.4%), Northern Ireland (5.9%), Scotland (2.0%).
- *Did not ask*: Wales (64.3%), England (51.9%), Scotland (42.9%), Northern Ireland (17.6%).

No significant differences or associations were found for age [*F* (1, 728) = 2.49, *p* = 0.59], parity [*χ* ^2^ = 5.63, *p* = 0.131] or ethnic group [*χ* ^2^ = 7.86, *p* = 0.548].

### Participants who did not enquire about milk donation

Three hundred and ninety‐one mothers were interested in donating their milk but assumed they would not be able to or did not realise at the time that they could. Participants were asked why they did not enquire about milk donation. Table 2 shows the proportion who selected each reason. The most common reasons included reading or being told that milk banks were too far away for milk to be collected, not realising that it was an option until too late and thinking that their baby was too old.

**Table 2:** Participants' reasons for not enquiring about milk donation (*n* = 391).

Reason	Strongly agree/agree	
	*N*	%
I read online/social media that milk banks were too far away	144	36.8
I was told by friends, family or peers that milk banks were too far away	99	25.3
I was told by a health professional that milk banks were too far away	79	20.2
I did not realise it was an option at the time	77	19.6
I thought my baby was too old	70	17.9
I thought I wouldn't be able to due to medication/health reason	50	12.8
I thought I wouldn't be able to due to lifestyle factors, e.g., alcohol intake	25	6.3
I had previously been told by a milk bank that it was not possible	17	4.3

Participants could add further reasons in an open‐ended box, and these included thinking that their milk had not been stored correctly, lack of freezer space, other people's difficult experiences or feeling the process was too difficult, being put off by minimum amounts, finding expressing difficult or not being able to afford an electric pump, finding early motherhood too challenging, preferring to give directly to mothers and being told by a health professional that pasteurisation destroys beneficial content.

### Participants who enquired but were told they were unable to donate

Two hundred and eighteen mothers were told that they could not donate milk (63.9% of those who enquired). The main reasons included distance from a milk bank, medication use, age of baby and volume of milk to donate (Table 3).

**Table 3:** Participants who enquired but were told they were unable to donate (*n* = 218).

Reason	Strongly agree/agree	
	*N*	%
I lived too far away from the milk bank	116	53.2
I was taking an unsuitable medication	85	38.9
My baby was too old	82	37.6
I didn't have enough milk to donate	82	37.6
My stored milk was expressed too long ago	21	9.6
Health reasons	18	8.2
My stored milk was not stored as per milk bank guidelines	14	6.4
Lifestyle reasons such as alcohol or smoking	7	3.2
I didn't have suitable storage facilities	6	2.7
I hadn't followed milk bank hygiene guidelines	4	1.8
I was told to keep my milk for my own baby as I was mixed feeding	4	1.8

Participants expanded on the reasons given in the open‐ended box. Some of these were logistical and had to do with milk bank capacity. This included stocks already being full (with some responses stating that they had ‘enough’ milk), a waiting list of donors or pause on recruitment due to staffing issues, and shortages of equipment such as blood collection vials for screening. Some women also experienced blood tests being paused during the Covid‐19 pandemic. Transport was often an issue with women living outside collection areas. Overall, 37 participants (16.9%) were unable to donate because the milk bank was at capacity or had too few staff to be currently accepting donations.

The age of the infant was a common barrier, with milk banks often requiring donors to start donating before their baby was 6 months old. However, significant variation was seen in the reported minimum age of the baby at sign‐up from 2 to 12 months. Logistical issues further complicated this. Low staffing numbers, time taken to conduct blood tests and then the time needed to express minimum volumes of milk meant that some women with babies who were 3 months old at the time were unable to be considered.

Another common issue reported was that milk could not be accepted that was stored in bags or sometimes syringes, particularly when babies had been admitted to the neonatal unit, rather than bottles. Other women were prevented due to having a secondhand pump, using a Haakaa‐style suction device or catching milk in milk shells (rather than directly expressing). It should be noted that these criteria, none of which are included in national donor recruitment guidance (National Institute for Health and Care Excellence [NICE] Clinical Guideline 93),^23^ vary between milk banks with current maximum recruitment at 24 months postnatally in two milk banks.

Participants who were not able to donate due to medications or procedures were asked to give details of which medications they were taking that were contraindicated. A pharmacist member of our team checked as to whether these medications are considered contraindicated for donation. We did not ask for specific dosage details and therefore only generalised information is given in Table 4. We split the medications into named (e.g., Citalopram) and generic (e.g., antihistamines). In some cases, some medications in a category such as antihistamines may be suitable when others are not. We considered whether medications were (a) compatible with a donation as there is evidence to support this, (b) incompatible because the recipient baby would need to be monitored which would not be possible or likely appropriate, (c) theoretically suitable but no data to confirm compatibility and therefore not able to be accepted, (d) suitable for donation but only for term babies and (e) temporarily unsuitable and would require interruption of donation until the drug has left the donating mother's breastmilk. As Table 4 shows, nine medications/procedures were listed that should be compatible with a donation with three further medications/procedures compatible with milk for term babies. Seven further medications would have been compatible if only used in the short term, meaning donation could be temporarily paused or delayed.

**Table 4:** Maternal medications and procedures that were seen as a barrier to donation.

	Medication	Current evidence/guidance	Category of drug
Compatible with donation	Acupuncture	No research on acupuncture during lactation has been located but assuming use by a qualified practitioner risk is assumed to be low. Further information needed	
	B12 injections	Assuming the dose prescribed by the GP, compatible with the donation	Vitamin deficiency
	Levothyroxine	Compatible with donation	Treatment of hypothyroidism
	Omeprazole	Compatible with donation	Proton pump inhibitor for reflux
	Paracetamol	Compatible with donation	Analgesic
	Metformin	Compatible with donation	Treatment of PCOS/diabetes
	Salbutamol	Compatible with donation	Treatment of asthma
	Nondrowsy antihistamines	Compatible with donation	Treatment of seasonal allergies, etc.
	Immunoglobulins	Compatible with donation as destroyed in the gut but specific medication information would be needed to clarify	Unclear from the data provided
Recipient baby would need to be monitored and therefore inappropriate for preterm infants but could potentially be used for term babies where the family is known.	Azathioprine	The recipient's baby would need to be monitored for potential side effects. Insufficient evidence to recommend use in donation	Immunosuppressant https://bnf.nice.org.uk/treatment-summaries/immunoglobulins/ant
	Hydroxycholoquine	The recipient baby may need to be monitored for symptoms of uveitis. Unlikely but insufficient evidence to recommend use in donation	Treatment of lupus/rheumatoid arthritis
	Levetiracetam (Keppra™)	Recipient's baby would need to be monitored for drowsiness. Insufficient evidence to recommend use in donation	Antiepilepsy
	Clonzepam	The recipient's baby would need to be monitored for drowsiness. Insufficient evidence to recommend use in donation	Antiepilepsy
	Labetalol	Potential to alter blood pressure and blood glucose levels of recipient baby. Insufficient evidence to recommend use in donation	Beta‐blocker (antihypertensive)
	Propranolol	Potential to alter blood pressure and blood glucose levels of recipient baby. Insufficient evidence to recommend use in donation	Beta‐blocker (antihypertensive)
	High blood pressure medication	Some acceptable in‐term babies, may alter blood pressure, and blood glucose or interact with the baby's own meds if preterm	Treatment of hypertension
	Mesalazine (Octasa™)	Could cause diarrhoea in the recipient baby. Insufficient evidence to recommend use in donation	Treatment of inflammatory bowel disease
	Olanzapine	Recipient baby would need to be monitored for drowsiness. Insufficient evidence to recommend use in donation	Treatment of bipolar
	Quetiapine	Recipient baby would need to be monitored for drowsiness. Insufficient evidence to recommend use in donation	Treatment bipolar
	Warfarin	Risk of bleeding in the baby. Insufficient evidence to recommend use in donation but rarely used in lactating women	Anticoagulant
	Ibuprofen	Currently under discussion. Widely used in the immediate postpartum period but current guidelines require milk for preterms is not collected for 12.5 h after the last dose. Suitable for full‐term babies	Analgesic
Theoretically suitable but no data to confirm compatibility	Adalimumab	It should not be absorbed from milk but no data on safety for preterm donation recipients	Immunosuppressants are used to treat rheumatoid arthritis, inflammatory bowel disease, etc.
	Dalteparin	It should not be absorbed from milk but no data on safety for preterms donation recipients and potential small risk of bleeding in recipient pre‐term baby, Suitable for donation to term babies	Anticoagulant
Suitable for donation for term babies	Citalopram	Suitable for donation to term babies	Antidepressant
	Escitalopram	Suitable for donation to term babies	Antidepressant
	Sertraline	Suitable for donation to term babies	Antidepressant
Temporary interruption of donation for 5 half‐lives of the drug after which it is assumed the drug has left the donating mother's breastmilk	Clindamycin	Pause donation during treatment and for 12 h after	Antibiotic
	Codeine	Pause donation during treatment and for 15 h after	Opioid analgesic
	Sumatriptan	Temporary interruption of donation 15 h	Migraine treatment
	Antibiotics for mastitis	Temporary interruption for 7.5 h after last dose (assuming flucloxacillin)	Antibiotic
	Antihistamines which cause drowsiness	Temporary interruption if used for 24 h. Can reduce breast milk supply if used long term	Allergic reaction
	Flucloxcillin	Temporary interruption for 7.5 h after the last dose	Antibiotic
	Antibiotics for long‐term urinary tract infection	Temporary interruption until 5 half‐lives after the drug has finished but long‐term use may make this impossible depending on the duration of the course	Prophylactic antibiotic
Very limited data thus incompatible	Naltrexone	No data on passage into breastmilk other than as a treatment for the treatment of opioid or alcohol dependency which would make breastmilk unsuitable for donation. Low‐dose naltrexone is used for other off‐label uses but has no data.	

Abbreviations: GP, general practitioners; PCOS, polycystic ovary syndrome; PPI, Proton pump inhibitor.

In terms of the health reasons given that prevented donation, some were classified as incompatible. For example, women were unable to donate because they were living with someone who smoked or vaped or their partner had sex with other men, as per the guidance from blood transfusion services and the current NICE Guideline on the operation of a human milk bank.^23^

However, other reasons given are not in line with preventing donation. For example, mothers reported treated anaemia, type I diabetes and hypothyroidism as reasons for incompatibility, but these should not be contraindications to becoming a milk donor, provided the donor reported that they were in good health. Ferrous sulphate, insulin and thyroid replacement therapies are not contraindications to donation. Others reported previous blood donations but receipt of blood transfusions is only a contraindication to donating in rare circumstances. Blood transfusions are not a complete contraindication, but donors need to delay their screening blood tests until up to 4 months after the most recent transfusion to ensure testing would take into account any seroconversion. Blood transfusions received prior to 4 months before donor screening serology testing are only a contraindication if having led to the prospective donor becoming designated as ‘at increased risk of developing Creutzfeld–Jakob disease [CJD]’, that is, having received blood/blood products from someone who went on to develop CJD or having received blood from more than 300 donors.^24^, ^25^ Similarly, in vitro fertilisation alone is not a contraindication.

### Participants who enquired and were eligible but decided not to donate

Fifty‐nine women decided not to donate after receiving further information (17.3% of those who contacted a milk bank). Table 5 shows the proportion who agreed with different reasons for deciding not to donate. Women often agreed with numerous reasons, with the most common being the process or transportation feeling too complicated, not liking blood tests, not wanting to take extra steps or worrying about doing it safely. A third felt that they were too busy to be able to follow the process. Participants expanded on reasons for not wishing to donate in an open‐ended box. Some of the main additional reasons included feeling the commitment was too much as expressing for them was painful, the logistics of getting milk to a milk bank, the difficulties of finding time to express, a partner worrying that the milk might be needed, cost implications of purchasing storage containers (or an assumption that they would have to purchase these themselves rather than being provided by the milk bank) and lack of freezer space.

**Table 5:** Participants who enquired and were eligible but decided not to donate (*n* = 59).

Reason	Strongly agree/agree	
	*N*	%
The logistics of getting my milk to a milk bank felt too complicated	53	89.8
My partner didn't want me to	33	55.9
It felt too complicated	33	55.9
I don't like blood tests	33	55.9
I didn't want to take extra steps such as sanitising my pump	30	50.8
I was worried about doing it safely	25	42.4
I was just too busy	23	38.9
I didn't want my milk to be heat treated	23	38.9
I couldn't express enough milk for the milk bank collection guidelines	20	33.8
I developed health reasons that led to me not being able to donate	20	33.8
I received negative comments from family	19	32.2
I decided to keep it as a backup for my own baby	17	28.8
I went back to work	17	28.8
I found lifestyle factors such as limiting alcohol intake too restrictive	16	27.1
I didn't have suitable storage/room in the freezer	15	25.4
I received negative comments from friends	13	22.0
I needed to take a medication considered unsuitable	11	18.6
My baby became too old	10	16.9
I thought I would be paid for my milk	10	16.9
I became pregnant again	9	15.6
I would only be able to donate for a short period of time	9	15.6
I decided I would rather give my milk directly to other women	9	15.6
My baby started needing more milk	7	11.8
My baby became unwell	7	11.8
I received negative comments from a health professional	7	11.8
I felt pressured to give it to a family directly	6	10.1

### Uses for milk that had already been expressed

Three hundred and eighty‐seven mothers described what they did with milk that they had already expressed (Table 6). Typically, mothers used it in more than one way each, but even when they used it in ways such as giving it to their baby or in cooking, 215 (55.5%) mothers also threw some remaining milk away. Additionally, 33 mothers threw milk away without giving any to their baby (8.5%). Other uses included making soap, giving it to their older toddler, donating to research, using it as a treatment for their own eczema and skin conditions and saving it for the next baby.

**Table 6:** Use for milk that had already been expressed (*n* = 387).

Use	N	%
Gave it to my baby	324	83.7
Threw it away	302	78.0
Used it in other ways e.g. in the bath/for infected eyes	285	73.6
Used it in cooking for my baby	265	68.5
It's still in the freezer	245	63.3
Gave it to another family	123	31.7
Turned it into mementoes such as jewellery	112	28.9

For those who gave milk away to another family (*n* = 123), 52 (42.2%) gave it to a family member, 76 (61.7%) gave it to a friend and 51 (41.4%) gave it to someone they did not know before giving their milk away. Overall, 72 mothers (58.5%) gave milk to more than one type of recipient, that is, giving it to both a friend and then again to someone they did not know. In terms of the participants who gave the milk to someone they did not know (*n* = 51), 37 (72.5%) donated the milk via the Human Milk for Human Babies Facebook group. Others advertised more broadly on social media, including in milk sharing, baby and feeding groups. Recipients included a same‐sex couple who went through surrogacy, adults with long Covid, a mother with a disabled child, a mother with breast cancer, a mother met on holiday who was struggling to feed and ‘random strangers off the internet’. This process typically involved delivering the milk to their home by car, it being collected from the home or meeting up at a mutually agreed location. Finally, participants were also asked if they had ever sold their milk. Three (2.4% of those who shared their milk) responded that they had sold it to another family and two to a private company.

## DISCUSSION

This study explored the reasons why women were unable to donate to a milk bank, including what happened to any milk that they had already expressed. Although some women would be contraindicated from donating milk due to health reasons, others who were prevented from donating could potentially do so with changes to infrastructure, greater investment in milk bank staffing and improved public health information. Given the known benefits for premature infant health and development,^1^, ^2^ reduced post‐discharge health costs^3^ and positive impact upon parental well‐being in receiving^5^, ^6^, ^7^, ^8^ and being able to donate milk,^16^, ^17^, ^18^, ^19^ our findings add to the case for further investment in human milk banking services. This is especially true given predictions that demand for DHM will rise,^12^ alongside our findings that many women discarded their milk if they could not donate it.

Current guidelines and research have typically focused on the provision of DHM for premature infants. However, it is likely that many more infants and parents could potentially benefit from its use, including infants admitted to neonatal or special care, those with infant hypoglycaemia and infants where supplementation is clinically indicated, such as lower initial milk supply after birth complications.^26^, ^27^, ^28^ Supplemental DHM is associated with increased breastfeeding on discharge,^10^, ^29^, ^30^, ^31^, ^32^ whereas formula supplementation is linked to earlier cessation of breastfeeding,^33^, ^34^, ^35^ increased risk of respiratory and gastrointestinal disease^36^ and may disrupt the infant microbiome.^37^ There are also potential negative implications for maternal mental health if mothers did not plan to or wish to supplement with formula milk.^38^ Understanding the barriers and potential modifiable factors to enabling more women to donate their milk and increase the supply available is therefore important.

Some differences in responses given emerged for demographic factors. Women with degree‐level education were more likely to enquire but be told that they could not donate whereas women with secondary education were the least likely not to enquire. This likely reflects increased awareness of milk banking, confidence to make the initial enquiry or perceptions of who considers themselves to be ‘someone who donates milk’ reflecting previous research on who is more likely to become a donor.^39^, ^40^ Differences in responses between countries may reflect different milk banking infrastructure. Women in Scotland were the least likely to receive no response and least likely to not enquire. Scotland has a national milk bank, Milk Bank Scotland, which is hosted by NHS Greater Glasgow and Clyde and covers the whole of Scotland. Conversely, there are currently no milk banks in Wales and there were no milk bank hubs there until 2022. Women from Wales were least likely to enquire and most likely not to get a response. This has implications for the development of other regional services such as in Wales. Wales and Scotland are perhaps more comparable (e.g., in terms of population size, devolved health care and a low number of health boards compared to England) making a regional service a potentially achievable target in Wales.

Although some mothers would not be able to donate their milk due to medication use, many reasons given for rejection are potentially modifiable. The first is the need for clearer information about donation criteria and processes being disseminated more widely at an earlier stage. A common experience was for mothers to approach milk banks when their infant was around six months or older, as they had found early motherhood overwhelming, by which point, depending on milk bank criteria, it was too late to register. Given the registration process can take several weeks, information could be given to breastfeeding mothers at their six‐week check or be included in antenatal information or hospital discharge packs. In one US‐based study, just 4% of mothers at a newborn check had discussed milk donation with a healthcare professional,^41^ although in another US study, around a third of mothers on the postnatal ward had heard of the concept of milk donation.^42^ We are not aware of research regarding awareness of milk donation in a UK population, and studies to explore the public's awareness of the concept of milk banking and the processes involved should be conducted.

Earlier promotion of the process of donation would also help to reduce factors that would prevent donation, such as how milk is stored. Anecdotally, different milk banks in the United Kingdom have different criteria for accepting milk expressed within breastmilk storage bags or previously expressed stashes of milk. Work is ongoing through the UK Association for Milk Banking to understand the diversity of screening and acceptance guidelines, with a view to creating an evidence‐based standardised approach. While there might be specific capacity or transportation pressures on milk banks, further work should be undertaken to ensure potential donors that cannot donate to one milk bank are sign posted to others that serve their area to prevent women from being unnecessarily excluded.

Investment into staffing and infrastructure should be another core priority. Over half of women who enquired were told they could not donate because they lived too far from a milk bank. While progress has been made in milk banks creating hubs for the collection of donated milk in areas of the United Kingdom without local milk banking services, long journey times still prevent some from donating.

Meanwhile, almost one‐fifth of our respondents did not receive a reply. Milk banks in the United Kingdom are often under‐staffed and resourced, opening for only a few hours a week, yet are responsible for donor recruitment, milk processing, storage and dispatch within this.^43^ It is not a surprise that less time can be spent on responding to donor enquiries than is optimal. One option could be to invest in a national centralised contact point for donors, with those who meet the criteria and wish to proceed being directed to their closest or most suitable milk bank, or banks with current capacity, reducing the need for individual milk banks to respond to all contacts. Updated national guidance that considers recipients' differing needs in addition to safety and quality assurance would also enable more mothers to donate or receive explanations as to why it is not possible. There is a responsibility for the care of women who enquire about milk donation, whether they are eligible or not.^44^

There is currently a paucity of published literature on the safety of medication use by milk donors. Given the most likely recipient will be extremely premature infants, there is a duty of care to exclude any theoretical risk of harm from milk that might have medication or metabolites. It is vital that milk banks err on the side of caution so as not to risk harm to infants, or loss of reputation to milk banks, which has taken a long time to re‐establish after most milk banks closed in the late 1980s as a result of human immunodeficiency virus.^45^ However, as indications for DHM expand to include more infants that are full term and healthy, or even for the treatment of older children and adults (e.g., with inflammatory bowel disease, burns), the level of risk decreases. More research is required to understand the safety and acceptability of giving milk from donors taking medications to older, healthy babies to understand what medications might be safe for this group.

Some women shared their milk with others when they could not donate it. Milk sharing has a long history, with multiple cultural and religious traditions based on the community support of a new mother, but social media may have increased wider sharing.^46^, ^47^ One concern is that the wider popularity of milk sharing might decrease the supply to milk banks for the most vulnerable infants.^48^ However, in one study of US and UK milk bank donors, although around half also shared milk with others, these mothers actually donated to the milk bank for a longer duration, and in the United States, they provided greater donation volumes.^39^ This may reflect feelings of positivity associated with providing more broad support for others.^49^, ^50^ In studies of women who share their milk with others outside of milk banking, one motivation for doing this is to reach those families who do not meet the criteria for milk bank support, suggesting they are a different group from milk bank donors.^51^, ^52^, ^53^, ^54^ Potentially these mothers may donate through a milk bank (and therefore milk bank procedures) if more families were able to access DHM from milk banks. Another study with milk‐sharing recipients in the United States found that families sought it for breast refusal, slow weight gain, latch complications due to tongue tie, formula intolerance, adoption or serious medical conditions.^55^ Recent UK research has shown the positive impact that receiving screened DHM in similar scenarios can have.^8^, ^56^

Some women described exchanging milk at home or in car parks to unknown families, which may carry safety risks, especially if advertised online. No one in our sample described sending milk by post, but in the United States, milk sent this way can arrive at a higher temperature than recommended^57^ and/or with high bacterial levels.^58^ Selling breast milk was rare, with just three mothers (2.4% of those who shared their milk) doing so, reflecting similar data that around 2% of milk donors sell their milk^39^ with women driven more by motivations to help others than financial reward.^59^, ^60^ However, purchasing milk online does occur, with most evidence from the United States,^61^ and may have potential complications. One US study found that up to 10% of human milk sampled from milk sold online was found to have cow's milk contamination, suggesting that milk was being supplemented with other species of milk to increase volume.^62^ Despite well‐recognised harms of the commercialisation of other biofluids,^63^, ^64^ there has been little discussion of the sale of human milk in UK regulatory spaces.^65^ Protection from these harms has been enshrined in the European Union Directive on Blood, Tissues and Cells (Directive 2004/23/EC), which states the human body should not be commercialised or the source of gain. The recent update to this Directive included DHM as a substance of human origin (SoHO). While the regulation of DHM currently differs between countries,^66^ there is a clear rationale for countries globally to legislate for DHM as a SoHO, with the aim of limiting the potential harms of commercialisation.^67^, ^68^

Further research could also explore perceptions of healthcare professionals to milk sharing in cases where the ‘milk sharer’ is clearly known to the participant and the risks minimal, for example, a breastfeeding relative, close friend or second mother (in a same‐sex partnership) who wishes to lactate. As far as we are aware, research has not explored this in a UK context, but anecdotal online discussions on milk‐sharing forums and professional groups highlight that difficult discussions arise when milk is shared within healthcare settings. Frequently shared milk is seen solely in terms of its risk, without the balance of considering the impact of an absence of human milk, breastfeeding continuation or impact upon maternal feeding preferences.^69^

It is also worth exploring whether any of the reasons for deciding not to donate milk after enquiry could be reduced with different information or support. Some of these factors are unlikely to change, for example, the additional logistical pressures of expressing (sterilising a pump, feeling it was too complicated, too busy). Personal preferences or anxieties such as disliking blood tests or restricting certain lifestyle factors will likely also always remain. However, other aspects may be modifiable. For example, of those who decided not to donate, over a third did not want to do so due to heat treatment of the milk. As above, when considering the potential impacts of medication, we must assume that most DHM goes to vulnerable premature infants and therefore exclude any theoretical risk of harm from contamination. Pasteurisation of milk is therefore important and although it does reduce immunological properties of breast milk,^70^ DHM still contains more immunological properties than infant formula, which does not contain these properties at all. This is reflected in research that shows reduced rates of health complications among infants given DHM over formula milk.^1^, ^2^ It would be useful to explore perceptions of this with potential donors as potentially some may believe that this process removes more protection than it does.

Finally, the role that others played in influencing decisions should be highlighted. A significant number of women who decided not to donate were dissuaded due to a partner's views (56%), friends' views (22%) or comments from a health professional (12%). Among those who did not enquire, it was common to base this on being told by others that milk banks were too far away. Although this may be true, it is possibly outdated information or applicable to an individual. Exploring these views is important as negative or unsupportive reactions from others may potentially be based on misunderstanding or a lack of awareness of the important role DHM can play for both infant health and parental well‐being.^6^, ^7^, ^8^ Women have previously described how family and friends not understanding their choice to donate or use DHM can feel difficult or distressing.^6^, ^8^ Although many healthcare professionals are supportive of the use of DHM, some hold reservations or questions around its composition, contamination or impact on donors.^15^, ^19^, ^71^, ^72^ We know that partners, family and friends, health professionals and the wider public can have important influences (both positive and negative) on women's infant feeding attitudes, decisions and experiences.^73^, ^74^, ^75^, ^76^ Awareness campaigns that improve public and professional knowledge about the use and benefits of DHM may help to change this and could be part of broader public conversations around the importance of breastfeeding and human milk.

Our study does have limitations. Participants were older than average with a higher level of education, although this may reflect who is able to consider donating milk. Mothers who breastfeed exclusively and for a longer duration^77^ are more likely to be older and have a higher level of education.^39^, ^40^ This may have been exacerbated by internet‐based recruitment, although the use of smartphones and social media in this age cohort is high and unlikely to restrict access any more than known limitations with face‐to‐face and hospital‐based recruitment. An alternative strategy may have been to collaborate with each milk bank in the United Kingdom to disseminate the survey to potential donors who were unable to donate. However, this would have (a) placed increased demand on services and (b) not reached those who felt unable to donate or who did not reach a response.

Women from White ethnic backgrounds were also overrepresented in our study. This might partly be explained by some mothers from Muslim families not wishing to donate (or receive) milk due to milk kinship: an Islamic belief that human milk creates a kinship between the breastfeeding mother's family, including her children, and the infants who receive her milk, which prevents marriage in Islamic law.^78^ This can be overcome by developing accurate systems to trace who has donated and received specific milk. Additionally, although historically all communities have practised wet nursing, there have also been periods when wet nursing has been used harmfully, such as forcing enslaved women to do so. This has implications within Black communities in relation to milk donation, exacerbated by recent events such as for‐profit milk banks in the United States specifically targeting Black mothers to sell their milk.^79^ Further culturally informed research is needed to explore and navigate these and other barriers to milk donation before true equity can be achieved.

To conclude, as more usage of DHM in UK neonatal units is predicted over the next 2 years, and ongoing trials in patient groups beyond extremely premature populations start to report, milk banking services are likely to require more women to donate their surplus milk. Further work will be needed to understand the proportion of families in which milk donation would be possible and acceptable and the volume of milk that may be donated as a consequence. Additional effort will be needed to understand how key messages about the prioritisation of breastfeeding their own babies, the optimal time for receiving messages about milk donation and modifications to screening and donation processes to ease the burden of time on milk donors. We also need to learn more about how milk donation can positively support and promote breastfeeding to achieve broader public health aims.

## AUTHOR CONTRIBUTIONS

Amy Brown was responsible for study conception, study design, data collection, data analysis and draft report writing. Sara Jones, Natalie Shenker and Gillian Weaver were responsible for study conception, study design and draft report writing. Wendy Jones and Catrin Griffiths were responsible for data analysis and draft report writing. All authors read and approved the final manuscript.

## CONFLICT OF INTEREST STATEMENT

Amy Brown coordinates the Swansea Human Milk Bank hub, a collaborative project between the Human Milk Foundation, Swansea University and Swansea Bay University Health Board, set up of which was funded through Research Wales Innovation Funding from HEFCW. This grant also funded the development of this research. Sara Jones has previously worked as an assistant co‐ordinator at the Swansea Human Milk Bank hub. Wendy Jones is an expert advisor in pharmacology to the Human Milk Foundation. Gillian Weaver is a cofounder and consultant for the Human Milk Foundation, a charity that operates the Hearts Milk Bank. Natalie Shenker is a cofounder of the Human Milk Foundation, a charity that operates the Hearts Milk Bank. She was a consultant for the Human Milk Foundation between October 2020 ‐ September 2024. She is a UKRI Future Leaders Fellow at Imperial College London (Grant p76489), and UKRI funding supported her participation in the writing of this manuscript. The remaining author declares no conflict of interest.

### PEER REVIEW

The peer review history for this article is available at https://www.webofscience.com/api/gateway/wos/peer-review/10.1111/jhn.13405.

## ETHICS STATEMENT

Ethical approval was granted by the School of Health and Social Care Research Ethics Committee, Swansea University.

## Data Availability

Data are available on request.
